# Study on Blending Modification of Bisphenol A Epoxy

**DOI:** 10.3390/polym15153263

**Published:** 2023-07-31

**Authors:** Xiaotao Fu, Long Ma, Lincong Chen, Cong Zhang, Xiaolin Chen, Xinran Li, Fangda Fu, Chuanfu Fu, Taobei Lin, Wensheng Mao

**Affiliations:** 1 Key Laboratory of Physical and Chemical Analysis for Electric Power of Hainan Province, Hairui Road No. 23, Haikou 570100, China; fuxiaotao123abc@163.com (X.F.); 13907640264@163.com (L.C.); zhangc_hn_dky@163.com (C.Z.); chenxiaol@hn.csg.cn (X.C.); lixinran@hn.csg.cn (X.L.); ffd@hn.csg.cn (F.F.); fuchuanfu@aliyun.com (C.F.); lintb@hn.csg.cn (T.L.); maows@hn.csg.cn (W.M.); 2 Hebei Key Laboratory of Green and Efficient New Electrical Materials and Equipment, North China Electric Power University, Yonghua North Street No. 619, Baoding 071003, China

**Keywords:** damp-heat aging, salt spray aging, epoxy resin, mixed system, electrical property, mechanical property

## Abstract

Epoxy-resin-based composites in the field of current electrical materials often work in high temperature, high humidity or salt spray conditions. In order to improve the operation reliability of the composite insulator cross arm in a high temperature, high humidity and high salt spray environment, and analyze the aging mechanism and performance characteristics of resin, in this paper, wet heat aging and salt spray aging experiments were carried out on the blended resin system composed of bisphenol A type epoxy resin (E-51), aliphatic epoxy modified bisphenol A epoxy resin (2021P/E-51) and dimeric acid modified bisphenol A epoxy resin (EPD-172/E-51). Among them, 10 wt% and 20 wt% of 2021P blend resin and 10 wt% of EPD blend resin have superior thermo-mechanical properties. Under humid and hot conditions, the dielectric loss of 10 wt% EPD blend system before and after aging is 39.9% and 49.5% lower than that of pure E51 resin system, respectively. Under the condition of salt spray, the dielectric loss of 20 wt% and 10 wt% EPD blends decreased by 73.1% and 74.6% after aging. The leakage current of 10 wt% 2021P blend resin and 10 wt% EPD blend resin decreased by 7% and 3.8% before aging, respectively. After aging, they decreased by 3.7% and 2.2%, respectively. The bending strength of 2021P blend resin before and after aging reached 29.3 MPa and 26.6 MPa, respectively. The above three blending resin systems exhibit good electrical properties and good mechanical properties, their ageing resistance performance is strong and they are suitable as the matrix resin of compound cross arm mandrel material.

## 1. Introduction

A composite cross arm is composed of glass-fiber-reinforced resin matrix composite mandrel, silicone rubber sheath and gold tools. Compared with the traditional iron cross arm, it can save a lot of steel, reduce the consumption of ore energy and has the advantages of good insulation performance, high strength and light weight. By the end of 2020, more than 30 pilot projects for compound cross arms had been completed in China [1]. The operation experience shows that the lightning trip rate of the distribution network line is greatly reduced, and the reliability of the system is obviously improved after the application of the composite cross arm. However, in the process of operation, the sealing property is often damaged due to pecking by birds and other reasons, and the mandrel is exposed to moist air, thus generating a partial discharge phenomenon, which further leads to the malignant consequence of a too-fast temperature rise and mandrel fracture. In addition, the glass-fiber-reinforced epoxy-resin-based composite insulated cross arm has low elastic modulus, relatively brittle and easy cracking, weak long-term temperature resistance, weak water resistance and easy-to-produce matrix plasticization and cracking in a high temperature, high humidity and high salt environment, which cannot meet the insulation requirements in harsh environment, thus affecting the stable operation of the system. In order to expand the application range of the composite cross arm and make it adapt to the high temperature, high humidity, high salt environment such as Hainan, higher requirements are put forward for the weatherability of composite cross arm. Therefore, improving the weatherability of composite cross arm core rod becomes an urgent problem to be solved.

In the field of high-voltage power transmission, especially in the distribution network of 10 kV voltage, the advantages of the epoxy resin composite cross arm are very obvious: it has light weight, high mechanical strength and good insulation performance, can improve the level of lightning resistance of the line and meet the requirements of insulation, so it has been widely used. Domestic and foreign experts have conducted a series of studies on the aging of epoxy resin matrix applied in the field of electric power energy. M. Alsaadi [2] explored the changes of mechanical properties of glass fiber/epoxy resin composites under the aging conditions of humid heat and ultraviolet light, and the results showed that the interfacial shear strength would decrease obviously after aging, and the adhesion of epoxy resin matrix of the composites was observed. Nie Zhangxiang et al. [3]. established an interface aging model of fiber-reinforced composites under hot and humid conditions and evaluated the aging resistance characteristics of the material interface through the aging rate of the interface image and the defect expansion index. Li Guangmao et al. explored the water absorption characteristics and failure mechanism of epoxy resin in hot and humid environment, proposed that the water absorption of epoxy resin meets the LangmuirEXT1 isothermal adsorption model and the saturated water absorption rate is a quadratic function of temperature and observed that the number of micropores after the water absorption aging of epoxy resin is positively correlated with the volume and aging temperature. After aging, the volume resistivity decreases rapidly, while the relative dielectric constant and dielectric loss angle increases, resulting in low-frequency dispersion [4]. Zhang Jin et al. explored the diffusion process of salt spray solution in resin. Through X-ray photoelectron spectroscopy analysis, they concluded that the deterioration of material properties was mainly caused by the diffusion behavior of water molecules and H^+^ and extracted water molecules produced swelling effect on the crosslinked network of resin polymers and generated stress at the interface to reduce the interface properties [5]. Douar M. A. [6] et al. compared the performance of insulators made of different polymers (polyamide, polycarbonate, aliphatic resins and EPDM elastomers) in the salt spray environment, and the results of AC flashover measurement and surface damage assessment showed that aliphatic epoxy resins were the most resistant to arc. TripathiG [7] et al. studied the blend toughening of bisphenol A type epoxy resin, carboxy-terminated butadiene-acrylonitrile and aliphicyclic epoxy resin and proposed that blends can significantly improve the physical and mechanical properties and heat resistance of epoxy system. Li Yue et al. Studied the corrosion behavior of carbon-fiber-reinforced epoxy resin composites under high temperature and high concentration of salt spray, explored its aging molecular mechanism and interface damage mechanism and proposed that the aging process can be divided into three stages, during which the swelling and plasticization of water molecules on the resin occur. The interface damage induced by the mismatch of fiber matrix wet expansion coefficient and the corrosion chain breaking effect of salt/water on the resin can lead to the reduction of cross-linking density of the system, interface debonding and matrix breakage, and induce the deterioration of material mechanics and interface strength [8]. Li Feng et al. Explored the aging properties of epoxy vinyl ester resin and its composites in mineralized water at high temperature and high pressure (70 °C + 5 MPa). The results showed that the characteristic functional groups of epoxy vinyl ester changed before and after aging, and physical and chemical reactions such as hydrolysis of ester group and formation of hydrogen bond inside the material occurred, which had adverse effects on the material properties [9].

In conclusion, the properties of matrix resin are mainly affected by heat and moisture aging and salt spray aging [10,11]. At present, bisphenol A epoxy resin, the most widely used in industrial production, has the advantages of high strength, good corrosion resistance and low cost, but its heat resistance and toughness are not high, and its humidity and heat resistance and weather resistance are poor. However, the preparation process of resins with good performance, such as aliphicyclic epoxy resin, is complicated, the production cost is high and the large-scale application is limited. Therefore, the blends of different kinds of resins are considered to reduce the cost and improve the performance of the resin matrix.

In this article, the low cost bisphenol A epoxy was blended with the high performance but high cost alicyclic epoxy, the properties of the blends of three matrix resins, bisphenol A epoxy (E-51), acyclic epoxy modified bisphenol A epoxy (2021P-51) and dimeric acid modified bisphenol A epoxy (EPD-172E-51), were studied under the conditions of hot-humid aging and salt spray aging. By comparing the bending strength and tensile strength of different resin systems before and after aging, the leakage current and breakdown voltage were tested and the effects of hygrothermal aging and salt spray aging on the mechanical and electrical properties of the matrix resin were analyzed to provide data support for the selection of epoxy systems in practical engineering applications.

## 2. Test Section

### 2.1. Test Material

The experimental materials used in this experiment included E-51 bisphenol A epoxy resin, aliphatic epoxy modified bisphenol A epoxy resin (2021P/E-51), toughened dimeric acid modified bisphenol A epoxy resin (EPD-172/E-51), curing agent methyl hexahydrophthalic anhydride (MHHPA), model DMP-30 epoxy curing accelerator, etc. [12,13]. Some properties of materials used in the experiment are shown in Table 1.

**Figure 1:**
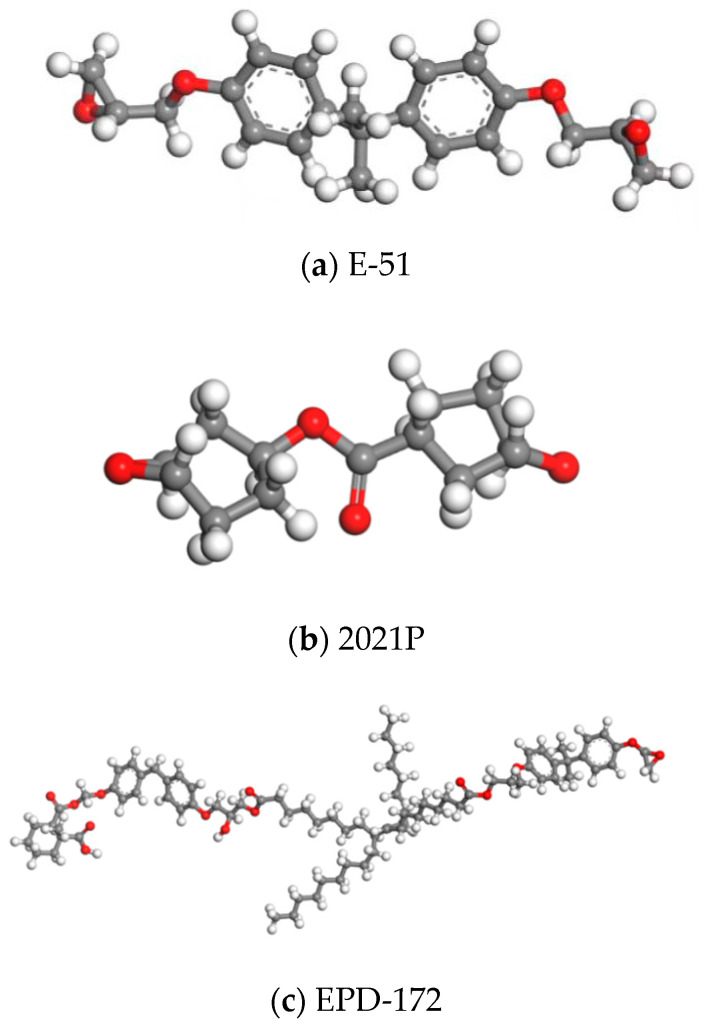
Molecular structure of resin.

The molecular structures of the three resins used in the experiment are shown in Figure 1a–c, respectively. The saturated six-membered ring in the type 2021P aliphatic epoxy resin replaces the benzene ring in E-51 and contains two aliphatic epoxy groups and one shrink ester group. It has higher epoxy value, lower viscosity and higher chemical stability [14]. On the basis of bisphenol A type resin, dimeric acid modified epoxy EPD-172 adds long chains of unsaturated fatty acids [15], and chain segments of unsaturated fatty acids enhance the toughness and viscosity of the resin, which is more conducive to its binding with fibers [16].

**Figure 2:**
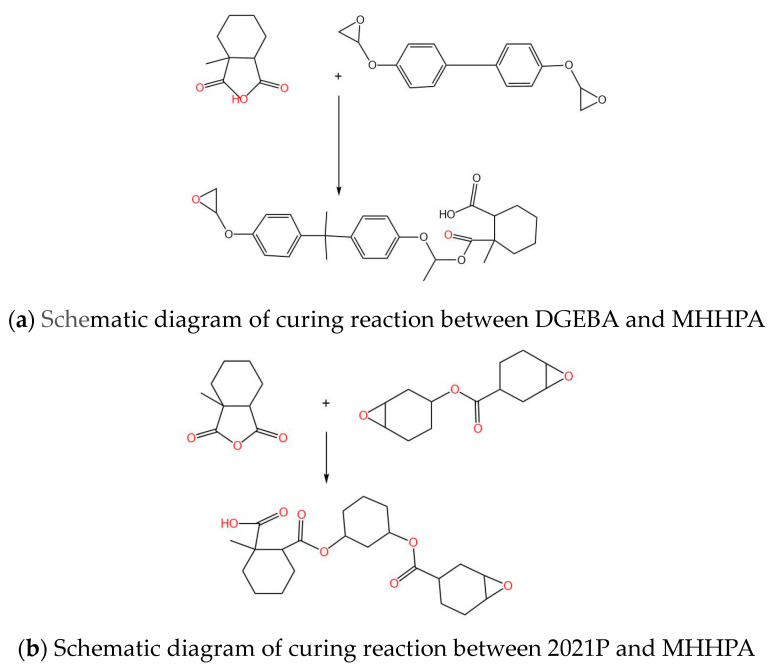
Schematic diagram of curing reaction.

Figure 2 shows a schematic diagram of the curing reaction of bisphenol A epoxy E-51 and 2021P.

### 2.2. Sample Preparation

In this study, two blending systems were set up. The overall properties of the blends increased first and then decreased with the increase of epoxy content. While the viscosity of EPD-172 was higher, a small amount of EPD-172 and E51 can ensure its good processability after blending, in order to meet the mandrel pultrusion, resin vacuum casting and other technological properties, comprehensive economic and technological factors, 2021P was mixed with E-51 epoxy resin, EPD-172 was mixed with E-51 epoxy resin according to the mass ratio of 10:0, 9:1, 8:2, 7:3, and then 80% of the total mass of the resin was added. Finally, the promoter DMP-30 with the mass fraction of 0.5% was added. Then we put the mixture into the planetary stirring/defoaming instrument, set the speed at 600 r/min, stirred for 120 s and removed some bubbles. The mixture was then further defoamed in a vacuum drying oven at 60 °C until there were no residual bubbles. The mixture was poured into the PTFE mold that had been brushed with release agent, the surface scraped carefully, placed in the oven for curing, heated at 140 °C for 10 h and the sample was taken out after the end of curing. Finally, 2021P/E-51 blended resin and EPD-172/E-51 blended resin were obtained.

The seven matrix resin blending systems obtained were numbered 1–7, as shown in Table 2. Among them, No. 1 is the pure E-51 resin system, No. 2, 3 and 4 are the blended resin system of E-51 and 2021P and No. 5, 6 and 7 are the blended resin system of E-51 and EPD-175. The electrical insulation and mechanical properties of No. 1–7 blended resin systems were tested respectively, from which the best ratio of matrix resin material that can improve the performance of composite cross arm core rod was selected.

Tensile and bending specimens were prepared to test the mechanical properties. The bending specimens were cut into cuboids with dimensions of 80 mm × 15 mm × 4 mm according to the standard ISO178 [17]. According to ISO527-2, the tensile sample was cut into a dumbbell shape with a middle width of 10 mm and a standard distance of 50 mm. After cutting the sample, the edges were sanded with fine sandpaper until smooth.

### 2.3. Test and Characterization

#### 2.3.1. Wet-Heat Aging Test

The hygrothermal aging test was carried out according to GB/T7141-2021. The epoxy samples were dried in an oven at 60 °C for 48 h and then boiled in a water bath at 100 °C. the conductivity of the water was adjusted to 1750 μsm by sodium chloride. The water absorption of the epoxy is mainly related to the difference in the concentration of the solution on both sides. Regular replacement of water to ensure that the insulator short sample is completely immersed in water can speed up the water absorption rate of the resin. Each group of samples selected 10 specimens. The wet-heat aging cycle was 14 days, after the completion of the wet-heat aging, a comprehensive comparison of the performance changes was performed.

#### 2.3.2. Salt Spray Aging Test

A salt spray aging test was carried out according to GB/T2423.17-2008. In order to better simulate the atmospheric environment in coastal areas, a neutral environment is adopted, and relative humidity is kept above 85%. Ten samples for each group of experiments were taken. After sampling, the sample was cleaned with deionized water to remove the influence of fog.

The ions and water in the salt spray environment permeate into the composite material, leading to the expansion and plasticization of the matrix, the desticking of the fiber–matrix interface and the weakening of mechanical properties. Under the combined action of temperature, water molecules, metal/non-metal ions, H^+^ and other factors, the resin matrix produces irreversible damage, which leads to the aging of the composite materials. In a hot and humid environment, water penetrates into the material, leading to an increase in dielectric constant and dielectric loss factor, causing local temperature rise and stress imbalance, and the matrix cracks under the action of stress imbalance for a long time [18]. In addition, part of the water molecules react with the material chemically, leading to the fracture and recombination of the main chain, resulting in the gradual occurrence of micro-cracks in the epoxy resin material [19]. With the constant increase of water absorption, the cracks continue to develop and eventually lead to the deterioration of the electrical and mechanical properties of the material, leading to the aging failure of the material.

#### 2.3.3. Electrical Performance Test

In this paper, the dielectric loss angle tangent test, breakdown voltage test and leakage current test are carried out.

The dielectric loss angle tangent test adopts YG9100 automatic anti-interference precision dielectric loss tester respectively at 1.5 kV, 2 kV, 2.5 kV and 3 kV to measure the dielectric loss factor before and after aging of dry samples of different mixed resin systems, and then calculate its average value. The test method was the Xilin bridge direct connection method, the experimental temperature was 25 °C and the test sample size was 34 mm × 54 mm × 2 mm.

The bearing capacity of the matrix resin to the electric field is reflected by the breakdown voltage. The stepwise step-up method is generally adopted to pressurize the sample in a spherical electrode with a diameter of 20 mm. The sample was sandwiched between the ball gaps, the voltage was increased at the rate of 1 kV/s until the sample was broken down and the breakdown voltage was recorded. In the process of voltage boost, the sample generates less heat in a short time, so the influence of chemical aging and thermal aging on the breakdown can be ignored, and the electrical breakdown is regarded as the main breakdown form of the sample. The breakdown strength is calculated as follows: E = U/d, E is the breakdown electric field strength, U is the breakdown voltage and d is the thickness of the sample. In order to prevent surface flashover, the test is carried out in a transparent glass container containing dimethylsilicone oil to ensure that the silicone oil does not exceed the sample 5–7 mm. The voltage at the time of breakdown was recorded. Three test pieces were selected for each group of samples, and five breakdown test points were taken for each test piece. A total of 15 power frequency breakdown voltage values were measured.

Leakage current testing is performed according to IEC62217-2012 standards. The leakage current of samples with different aging periods is required to be measured [20]. The voltage was boosted to 12 kV at a constant rate of 1 kV/s [21], the current value was measured with a digital multimeter DM-3068, the current accuracy was ±0.001 A and the test sample size was 34 mm × 54 mm × 30 mm.

#### 2.3.4. Mechanical Property Test

This experiment needs to test the tensile and bending properties of the sample. In the experiment, the maximum tensile stress received by the sample until it breaks is the tensile strength, and the maximum bending stress that the material can bear when it breaks under the bending load or reaches the specified deflection is the bending strength. The model of mechanical test is a universal mechanical tester [22]. The tensile test is in accordance with the requirements of ISO 527-2-2012, the width of the middle of the specimen is 10 mm, the gauge is 50 mm, the loading rate is 5 mm/min, the bending test is in accordance with the requirements of ISO 178-2010, the specimen size is 80 mm × 15 mm × 4 mm, the span is 64 mm and the loading rate is 2 mm/min. Ten samples for each type are taken, and the mean value is calculated after removing the maximum and minimum values [23].

## 3. Test Results and Analysis

### 3.1. Electrical Insulation Property

#### 3.1.1. Dielectric Loss Tangent

Insulating materials in the electric field due to dielectric conductivity and dielectric polarization hysteresis affect internal energy loss, generally using dielectric loss factor to measure the degree of dielectric loss. When the dielectric loss is too high, material heating and cracking may occur, which affects the long-term safe and stable operation of the device.

**Figure 3:**
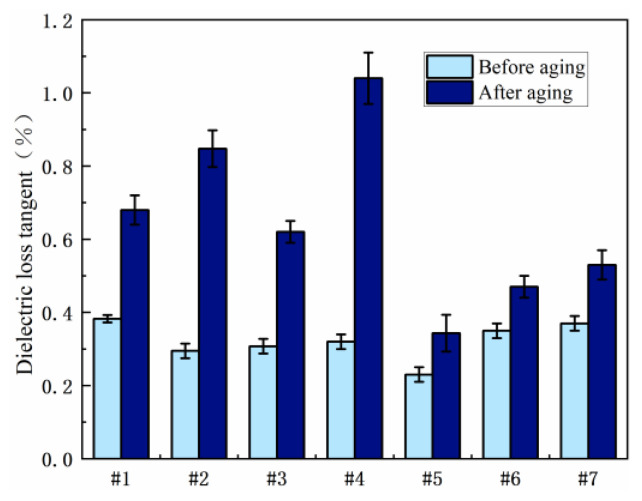
Comparison of dielectric loss of resin systems before and after hygrothermal aging.

Figure 3 shows the experimental results of dielectric loss before and after the wet and thermal aging of the resin system. It can be seen from the experimental results that the dielectric loss angle will increase before and after the aging of the epoxy resin system. It also increases the electrical conductivity of the material, so the dielectric constant and dielectric loss value gradually increase with the increase of water content.

In general, the dielectric loss of 2021P blends was low before aging, and the change of dielectric loss after hygrothermal aging showed a trend of first decreasing and then increasing with the increase in 2021P resin content, that is to say, with the increase of 2021P content, the anti-damp-heat aging property increased first and then decreased. The dielectric loss angle tangent of resin system No. 2 is 0.62, and that of resin system No. 1 is 0.68, compared with pure E51 resin. The dielectric loss tangent of 20 wt% 2021P blend resin system decreased by 8.8% after hygrothermal aging. This is because the epoxy group of 2021P is directly on the alicyclic ring, the molecular rigidity is greater and the structure is stable after crosslinking, so the dielectric loss caused by the turning polarization is smaller. The results showed that the dielectric properties of the blends were improved by the use of alicyclic epoxy, and the dielectric properties of the blends were better when the mass fraction of 2021P was about 20%.

The increase in the dielectric loss value of the EPD blend system was lower than that of the 2021P blend system and the pure E51 resin system. The dielectric loss of the blends with 10 wt% EPD resin decreased by 39.9% and 49.5%, respectively, compared with that of the pure E51 resin. This is because the molecular structure of EPD resin contains a large number of benzene ring structures. A benzene ring structure has a larger steric hindrance effect, which can limit the carrier migration in the resin matrix and reduce conductivity loss, therefore, it can enhance the resistance to damp-heat aging, so the dielectric loss value will be reduced, and the swing of the long carbon chain in EPD will increase the polarity loss and will increase the dielectric loss value. The dielectric loss increases with the increase of EPD content.

**Figure 4:**
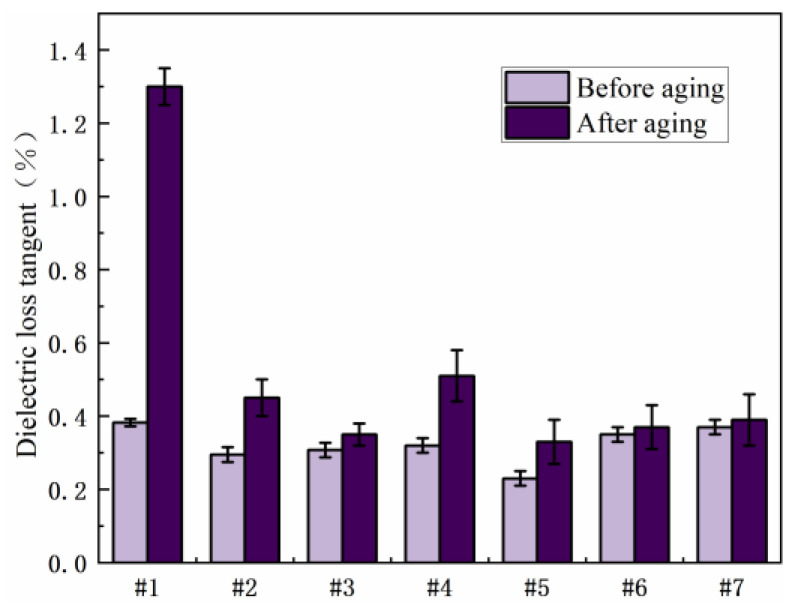
Comparison of dielectric loss of different resin systems before and after salt spray aging.

In the salt spray environment, the water molecules and ions in the resin matrix exist in the form of ionic conductance. With the invasion of salt spray, the polarization level of the interface increases. At this time, the electrons and ions in the electric field will accumulate at the heterogeneous interface, so that the different sign charges will be distributed on the surface of the adjacent electrode, causing leakage current loss. Figure 4 shows the experimental results of dielectric loss before and after salt spray aging. As shown in Figure 3, compared with the pure E-51 resin system, the dielectric loss values of the blended system with 2021P and EPD after salt spray aging are significantly reduced, and the anti-salt spray aging performance is significantly enhanced. Among them, the dielectric loss of No. 3 and No. 5 has the most obvious decrease, which is 73.1% and 74.6%, respectively, compared with the pure E-51 resin system, showing very good resistance to salt spray aging.

In the blending system studied in this paper, the main factor of dielectric loss occurs when polar molecules turn to polarization. On the aliphoring, the molecular rigidity is relatively large, and the structure is stable, which reduces the dielectric loss caused by the turning polarization. Therefore, adding a certain amount of aliphoring epoxy resin 2021P can improve the dielectric property of the blend system.

#### 3.1.2. The Breakdown Voltage

The voltage resistance tests of different epoxy resin systems before and after aging were carried out, and the power frequency breakdown field strength was measured according to the Weibull distribution function recommended by IEEE standard. The two-parameter Weibull cumulative distribution function is related to the sample breakdown probability when the power frequency field intensity is *E*. The probability *F*(*E*) of sample breakdown is:

(1) $$F(E)=1-\exp[-{\left(\frac{E}{E_{0}}\right)}^{\beta }]$$

In the formula, *E*_0_ and *β* are scale parameters and shape parameters respectively, both of which are positive values. *E*_0_ is used as the breakdown field strength of the sample. The abscissa is the breakdown field strength, and the ordinate is the breakdown probability. The 63.2% breakdown field strength is the average of the breakdown field strength.

**Figure 5:**
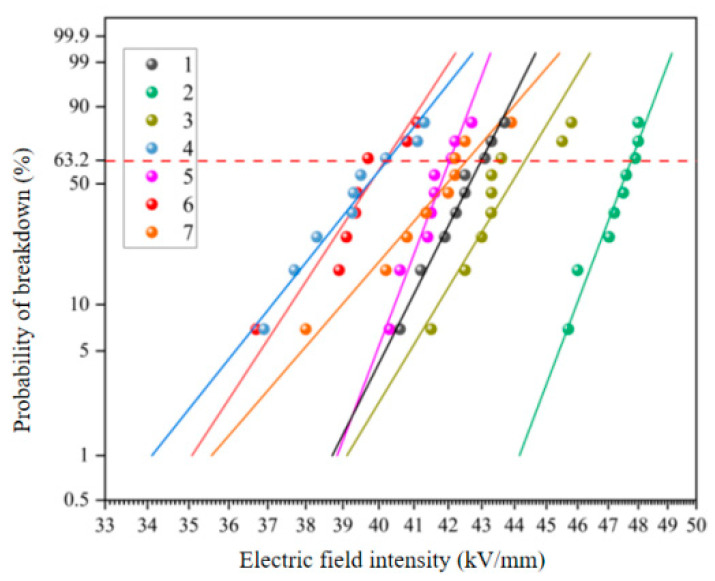
Weibull distribution of breakdown voltage of resin systems before hygrothermal aging.

Figure 5 shows the power frequency breakdown experiment of each resin system before aging, which can be obtained by Weibull statistical analysis. Under the breakdown probability of 63.2%, the corresponding breakdown field strengths of samples 1–7 are 42.99 kV/mm, 47.76 kV/mm, 44.33 kV/mm, 40.24 kV/mm, 41.56 kV/mm, 40.18 kV/mm and 42.55 kV/mm, respectively. As can be seen from the figure, the breakdown field strength of 2021P blend resin added 10 wt% before aging increases most obviously, by 10.7%. The breakdown field strength of 2021P blend resin system sample decreases to a certain extent with an increase in 2021P resin content. For the EPD blending system, the electrical strength of sample 5 and 7 is similar, while the breakdown field strength of sample 6 is relatively low.

**Figure 6:**
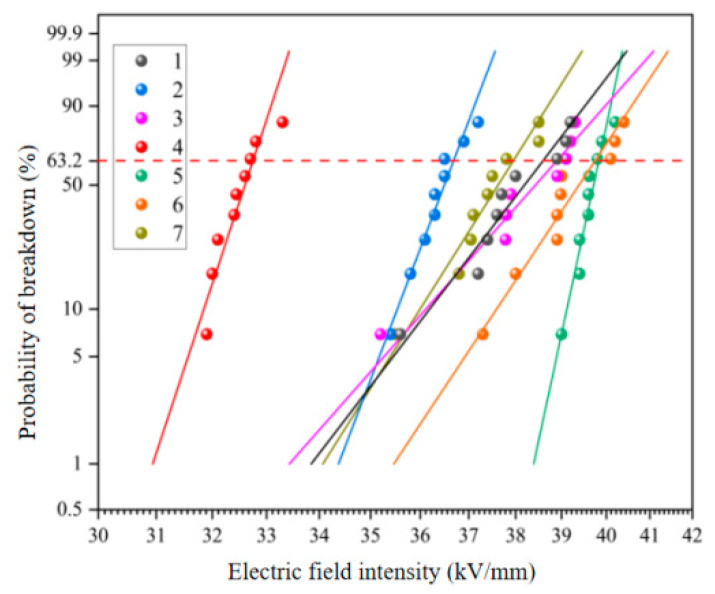
Weibull distribution of breakdown voltage of resin systems after hygrothermal aging.

Figure 6 shows the results of the power frequency breakdown experiment of each resin system after damp-heat aging. As can be seen from the figure, according to Weibull statistical analysis, when the breakdown probability is 63.2%, the breakdown field strength of samples 1–7 is 38.58 kV/mm, 36.68 kV/mm, 38.89 kV/mm, 32.74 kV/mm, 39.83 kV/mm, 39.74 kV/mm, 37.94 kV/mm. After wet and heat aging, the breakdown field strength of No. 5, No. 6 and No. 3 blends decreased less than before aging, and the electrical insulation strength remained at a high level. Based on the molecular structure analysis of the selected resin, because EPD resin has a benzene ring structure and 2021P resin lacks a benzene ring structure, the steric hindrance effect of the benzene ring effectively limits the carrier migration of the resin. To a certain extent, the electrical strength of the resin system can be enhanced, so the overall anti-aging ability of EPD blend system is better than 2021P blend system.

**Figure 7:**
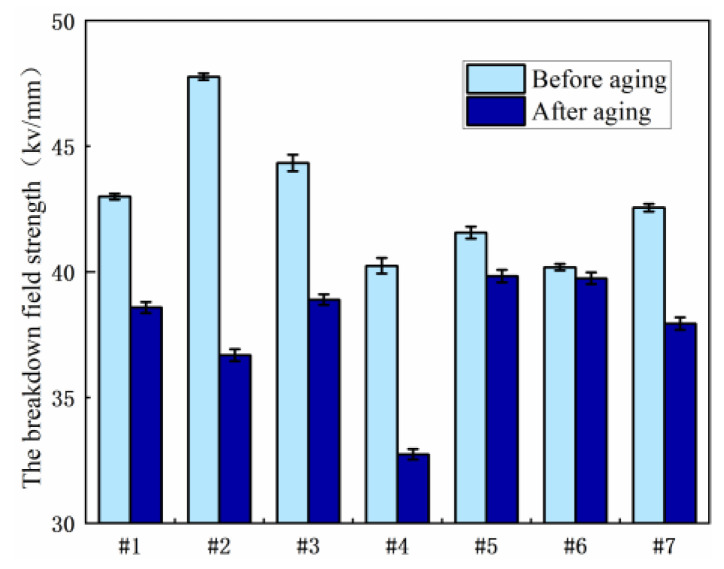
Comparison of breakdown field strength of various resin systems before and after hygrothermal aging.

Figure 7 shows the results of the breakdown field strength test of each resin system before and after hydrothermal aging. It can be seen from the experimental results that the breakdown field strength of each resin system will be reduced to varying degrees after aging. The breakdown field strength of sample No. 2 is the highest before hygrothermal aging, but it decreases sharply after hygrothermal aging; the reduction of No. 5 and No. 6 samples after hydrothermal aging is less than that before aging. Generally speaking, No. 3, 5 and 6 samples are more applicable in high temperature and high humidity environments.

**Figure 8:**
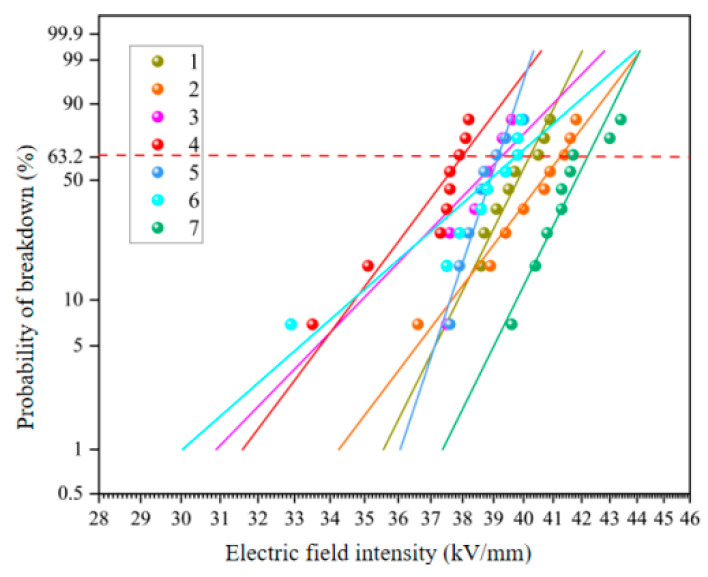
Weibull distribution of breakdown field strength of various resin systems after salt spray aging.

Figure 8 shows the results of the power frequency breakdown experiment of each resin system after salt spray aging. According to the Weibull distribution, the electric field intensity of each resin system is 40.19 kV/mm, 41.24 kV/mm, 39.25 kV/mm, 37.97 kV/mm, 39.14 kV/mm, 39.72 kV/mm and 42.20 kV/mm, respectively, under the breakdown probability of 63.2% after aging of salt spray. After wet and thermal aging, the electrical insulation strength of samples No. 7 and No. 2 remained at a high level, and the breakdown field strength was higher, respectively 2.6% and 5.0% higher than that of the pure E51 resin system.

**Figure 9:**
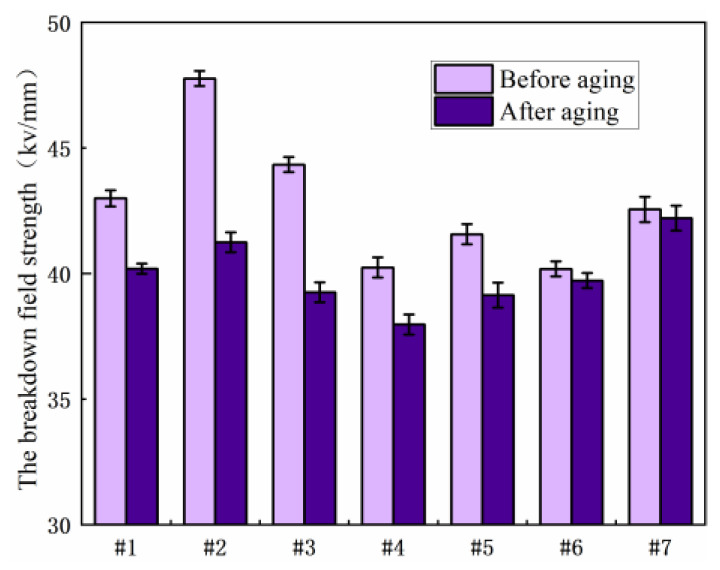
Comparison of breakdown field strength of various resin systems before and after salt spray aging.

Figure 9 shows the results of the breakdown field strength test of each resin system before and after salt spray aging. It can be seen from the experimental results that the breakdown field strength of sample 2 before salt spray aging is the highest, and the breakdown field strength after salt spray aging is second only to that of sample 7; after salt spray aging, sample No. 7 decreased less than that before aging, so sample No. 2 and sample No. 7 were more applicable in a high-salt environment.

The introduction of 2021P epoxy resin increased the crosslinking density of the blend system, and the intermolecular gap was small, which made it difficult to crack and break down [24], so the breakdown voltage of the blend system increased. With an increase in the content of aliphatic epoxy resin, the dielectric loss factor of the blending system decreases first and then increases, and the insulation resistance increases first and then decreases with the introduction of aliphatic epoxy resin. Therefore, under pressure conditions, the breakdown voltage will be higher when the dielectric loss of composite material is smaller, and the insulation resistance is larger [25]. Therefore, the breakdown voltage value of resin blending system needs to consider the above two aspects.

#### 3.1.3. Leakage Current

As an important indicator of dielectric insulation, leakage current can be used to reflect the overall dampness and deterioration of dielectric.

**Figure 10:**
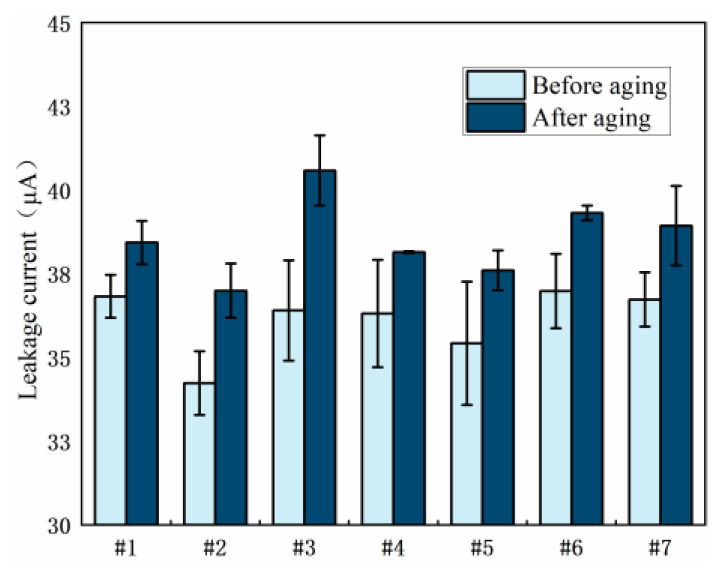
Comparison of leakage current of resin systems before and after hygrothermal aging.

Figure 10 shows the leakage current of seven resin systems before and after damp-heat aging. It can be seen from the figure that, before aging, as the content of 2021P and EPD-172 resins increases, the leakage current initially decreases and then increases. Samples 2 and 5 exhibit lower leakage currents and show a reduction of 7.0% and 3.8%, respectively, compared to the pure E51 resin system, indicating better performance than other resin blends. After aging, samples 2 and 5 still maintain low levels of leakage current, exhibiting reductions of 3.7% and 2.2%, respectively, compared to the pure E51 resin system, demonstrating good resistance to wet-heat aging. In conclusion, based on the leakage current test results, the performance of the resin systems with 10 wt% added 2021P blend resin and 10 wt% added EPD-172 blend resin is superior to that of the pure E51 resin system.

**Figure 11:**
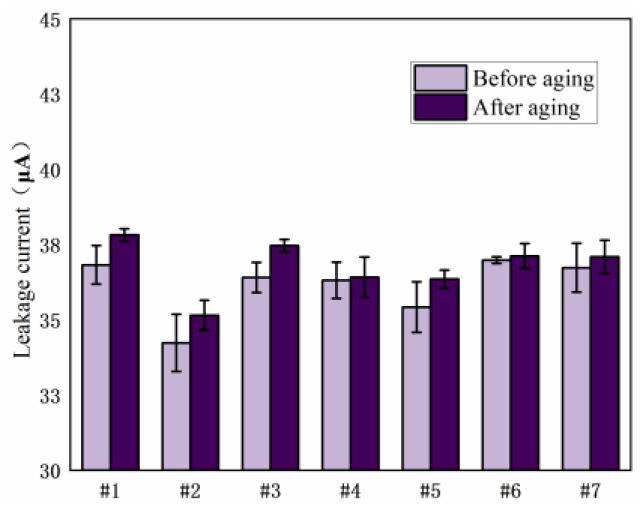
Comparison of leakage current of resin systems before and after spray aging.

Figure 11 shows the leakage current of each resin system after salt spray aging. It can be seen from the figure that, before and after aging, the leakage current generally shows a decreasing and then increasing trend with an increase in the content of 2021P and EPD-172 resins. Among them, the samples with 10 wt% added EPD-172 blend resin and 10 wt% added 2021P blend resin exhibit the lowest leakage current, outperforming other resin compositions, indicating good electrical performance and resistance to salt spray aging.

### 3.2. Mechanical Properties

#### 3.2.1. Bending Characteristics

**Figure 12:**
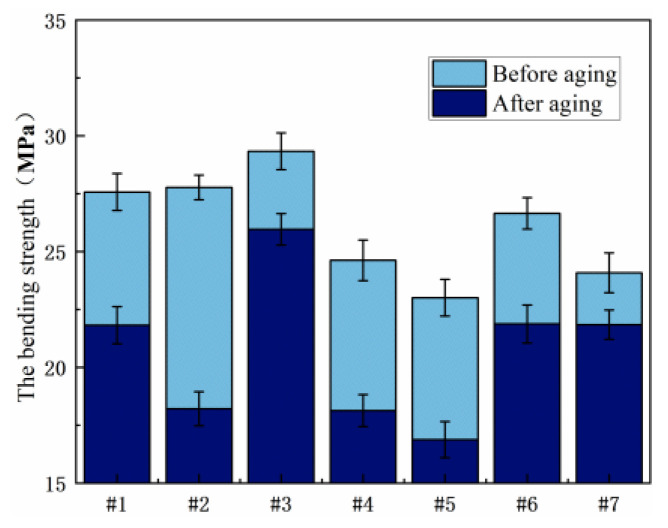
Comparison of bending properties before and after hygrothermal aging.

Figure 12 shows the comparison of flexural strength of seven resin systems before and after damp and heat aging. As can be seen from the figure, the mechanical properties of No. 3 resin are the best, and the bending strengths before and after aging are 29.33 MPa and 26.59 MPa, respectively, higher than those of other ratios. In the EDP and E51 resin blend system, No. 6 has excellent mechanical properties and maintains high bending strength after wet heat aging.

**Figure 13:**
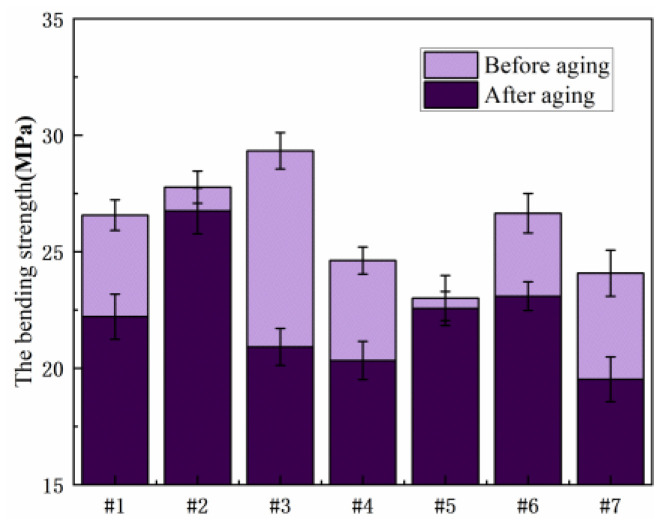
Comparison of bending strength of different resin systems after salt spray aging.

Figure 13 shows the comparison of bending strength of seven resin systems before and after salt spray aging. The bending strength of No. 3 and No. 6 is higher, and the reduction in bending strength after aging is lower than that of other blends. Salt spray aging resistance is good.

#### 3.2.2. Tensile Characteristic

**Figure 14:**
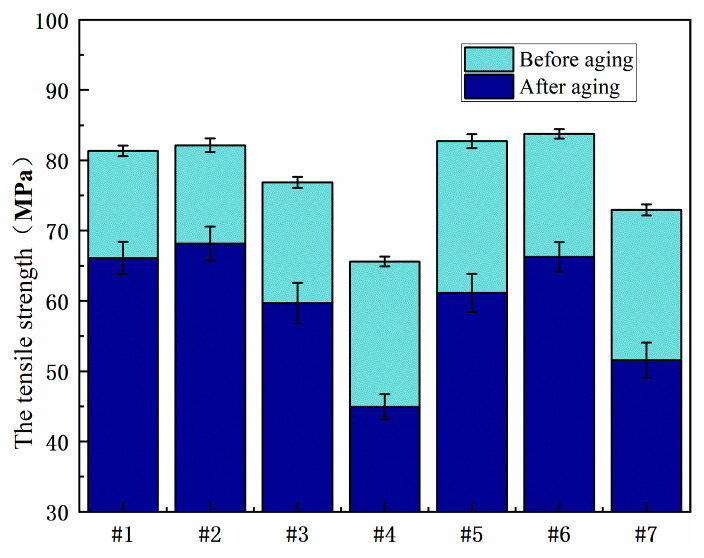
Comparison of tensile strength of resin systems before and after hygrothermal aging.

Figure 14 shows the comparison of the tensile strength of the resin system before and after moisture-heat aging. The tensile strength of the resin system after moisture-heat aging first increases and then decreases with the increase in the resin content in 2021P, but with the increase in the resin content in 2021P, the tensile strength of the blend system decreases. No. 2, No. 5 and No. 6 have higher tensile strength before and after aging, showing better mechanical properties.

**Figure 15:**
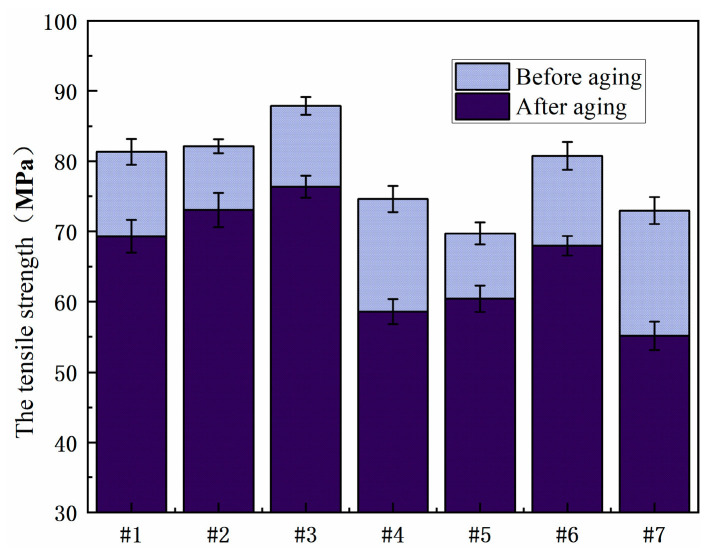
Comparison of tensile strength of different resin systems after salt spray aging.

The comparison of tensile strength of each resin system after aging with salt spray is shown in Figure 15. No. 2, No. 3 and No. 6 still maintain high tensile strength after salt spray aging, showing better mechanical properties. The bending strength of No. 2 and No. 5 aging is reduced less, and the resistance to salt spray aging is better.

The bending and tensile properties of the resin system are related to the strength of the matrix [26]. When the resin matrix is subjected to moisture and heat and salt spray, H_2_O, Na^+^ and Cl^−^ are diffused in the matrix [27], which increases the plasticity, decreases the stiffness and decreases the strength of the resin matrix [28]. Through analysis, it was found that the molecular chain has strong rigidity by directly connecting the epoxy group provided by the aliphicyclic epoxy resin [29,30], and the rigidity of the molecular chain segment can be enhanced by appropriate introduction of 2021P epoxy resin. However, because the molecular structure of 2021P is relatively clustered, it is not easy to form long chains. When the added 2021P content exceeds the critical value (mass fraction is 20%), the brittleness of the blended resin system increases, and the bending and tensile properties decrease.

## 4. Conclusions

- Adding a certain amount of alicyclic epoxy resin or toughened dimer acid to modify bisphenol A epoxy resin can improve the bending strength and tensile strength of bisphenol A epoxy resin system and improve its mechanical properties, but too much alicyclic epoxy or toughened dimeric acid modified bisphenol A epoxy can degrade its mechanical properties.
- The addition of alicyclic epoxy resin and toughened dimeric acid modified bisphenol A epoxy resin can reduce the leakage current and dielectric loss of bisphenol A epoxy resin system, improve its penetration resistance and improve the electrical insulation performance of the resin system. On the whole, the electrical insulation performance of 2021P blend system before aging was better than that of EPD blend system. The breakdown field strength and leakage current of 2021P blend resin with 10 wt% additive were significantly better than those of other samples before aging, however, the electrical properties of the resin system decreased with an increase in the epoxy content, and the electrical insulation performance of the aging EPD system was better than that of the 2021P system as a whole. The electrical insulation performance of EPD blend resin with 10 wt% additive is the most stable after aging.
- By comparing the electrical and mechanical properties of the seven mixed resin systems before and after wet heat aging and salt spray aging, the dielectric and electrical properties of No. 3 and No. 5 are better, the bending properties of No. 3 and No. 6 are better and the tensile properties of No. 2 and No. 6 are better. All in all, it can be seen that 2021P blend resin with 10 wt% and 20 wt% addition and EPD blend resin with 10 wt% addition have superior comprehensive performance and strong aging resistance, which is suitable for the research and development of new composite materials.
- Considering economic and other factors comprehensively, EPD172 and BFRP-2021P are used as composite insulating cross arm core rod materials in harsh climates, such as high humidity and salt, which have broad application prospects.

## Figures and Tables

**Table 1:** Chemical properties of experimental materials.

Materials	Molecular Weight	Density (g/cm^3^)	Source
E-51	375.86	1.22	Pelim Electric Technology Co., Ltd., Quzhou, China
2021P	252.31	1.17	Daicel Corporation, Osaka, Japan
EPD-172			LOHO HIGH-TECH, Shanghai, China
MHHPA	168.19	1.16	Guangzhou Desheng Chemical Co., Ltd., Guangzhou, China
DMP-30	265.40	0.97–0.99	

**Table 2:** Proportions of resin mixing system.

Sample	E51/wt%	2021P/wt%	EPD-172/wt%
1	100	0	0
2	90	10	0
3	80	20	0
4	70	30	0
5	90	0	10
6	80	0	20
7	70	0	30

## Data Availability

Not applicable.
